# A type VII secretion system of *Streptococcus gallolyticus* subsp. *gallolyticus* contributes to gut colonization and the development of colon tumors

**DOI:** 10.1371/journal.ppat.1009182

**Published:** 2021-01-06

**Authors:** John Culver Taylor, Xinsheng Gao, Juan Xu, Michael Holder, Joseph Petrosino, Ritesh Kumar, Wen Liu, Magnus Höök, Chris Mackenzie, Andrew Hillhouse, Wesley Brashear, Maria Patricia Nunez, Yi Xu

**Affiliations:** 1 Center for Infectious and Inflammatory Diseases, Texas A&M Health Science Center Institute of Biosciences of Technology, Houston, Texas, United States of America; 2 Center for Metagenomics and Microbiome Research, Baylor College of Medicine, Houston, Texas, United States of America; 3 Department of Microbiology and Molecular Genetics, McGovern Medical School, UT Health, Houston, Texas, United States of America; 4 Texas A&M Institute for Genome Sciences and Society, Texas A&M, Texas, United States of America; 5 Department of Microbial Pathogenesis and Immunology, College of Medicine, Texas A&M Health Science Center, Texas, United States of America; Columbia University, UNITED STATES

## Abstract

*Streptococcus gallolyticus* subspecies *gallolyticus* (*Sgg*) has a strong clinical association with colorectal cancer (CRC) and actively promotes the development of colon tumors. However, the molecular determinants involved in *Sgg* pathogenicity in the gut are unknown. Bacterial type VII secretion systems (T7SS) mediate pathogen interactions with their host and are important for virulence in pathogenic mycobacteria and *Staphylococcus aureus*. Through genome analysis, we identified a locus in *Sgg* strain TX20005 that encodes a putative type VII secretion system (designated as *Sgg*T7SS^T05^). We showed that core genes within the *Sgg*T7SS^T05^ locus are expressed *in vitro* and in the colon of mice. Western blot analysis showed that *Sgg*EsxA, a protein predicted to be a T7SS secretion substrate, is detected in the bacterial culture supernatant, indicating that this *Sgg*T7SS^T05^ is functional. Deletion of *Sgg*T7SS^T05^ (TX20005Δ*esx*) resulted in impaired bacterial adherence to HT29 cells and abolished the ability of *Sgg* to stimulate HT29 cell proliferation. Analysis of bacterial culture supernatants suggest that *Sgg*T7SS^T05^-secreted factors are responsible for the pro-proliferative activity of *Sgg*, whereas *Sgg* adherence to host cells requires both *Sgg*T7SS^T05^-secreted and bacterial surface-associated factors. In a murine gut colonization model, TX20005Δ*esx* showed significantly reduced colonization compared to the parent strain. Furthermore, in a mouse model of CRC, mice exposed to TX20005 had a significantly higher tumor burden compared to saline-treated mice, whereas those exposed to TX20005Δ*esx* did not. Examination of the *Sgg* load in the colon in the CRC model suggests that *Sgg*T7SS^T05^-mediated activities are directly involved in the promotion of colon tumors. Taken together, these results reveal *Sgg*T7SS^T05^ as a previously unrecognized pathogenicity determinant for *Sgg* colonization of the colon and promotion of colon tumors.

## Author summary

Colorectal cancer (CRC) is a leading cause of cancer-related death. The development of CRC can be strongly influenced by specific gut microbes. Understanding how gut microbes modulate CRC is critical to developing novel strategies to improve clinical diagnosis and treatment of this disease. *S*. *gallolyticus* subsp. *gallolyticus* (*Sgg*) has a strong clinical association with CRC and actively promotes the development of colon tumors. However, the specific *Sgg* molecules that mediate its pro-tumor activity are unknown. Here we report the first characterization of a type VII secretion system (T7SS) in *Sgg*, designated as *Sgg*T7SS^T05^. We further demonstrate that *Sgg*T7SS^T05^-mediated activities are important for *Sgg* to colonize the colon and to promote the development of colon tumors. These findings reveal *Sgg*T7SS^T05^ as a novel pathogenicity determinant of *Sgg* and provide a critical breakthrough in our efforts to understand how *Sgg* influences the development of CRC. Future investigations of the biological activities of specific effectors of *Sgg*T7SS^T05^ will likely lead to the discovery of *Sgg* molecules that can be used as diagnostic markers and intervention targets aimed at mitigating the harmful effect of *Sgg*.

## Introduction

Colorectal cancer (CRC) is the third most common cancer among men and women, and a leading cause of cancer-related deaths worldwide [1]. The ability of the gut microbiota to influence cancer risks and to modulate the development of CRC is well recognized [2–8]. The gut microbiota of CRC patients often displays distinct compositional differences and altered diversity compared to those from healthy individuals [8–11]. Furthermore, certain bacterial species are able to promote the development of colon tumors in pre-clinical models of CRC [2,3,5,6,12–14]. This has raised the possibility that by targeting specific CRC-promoting microbes, we can improve the way CRC is diagnosed, treated and managed. To achieve this goal, understanding the molecular mechanism underlying the cancer-promoting capability of specific microbes is critical.

*Streptococcus gallolyticus* subspecies *gallolyticus* (*Sgg*), previously known as *Streptococcus bovis* biotype I, is a member of the *Streptococcus bovis/Streptococcus equinus* complex [15]. *Sgg* is an opportunistic pathogen that causes bacteremia and infective endocarditis (IE). It is also known for its strong clinical association with CRC, as documented by numerous case reports and studies over the past several decades [12,16–28]. A study of case series and reports published from 1970 to 2010 found that on average, ~60% of patients with *Sgg* IE/bacteremia have concomitant colon adenoma or adenocarcinoma, a rate much higher than that in the general population [19]. In a prospective study, a higher percentage of patients with *Sgg* IE developed colonic neoplastic lesions in subsequent years compared to patients with IE caused by closely related enterococci (45% vs. 21%) [17]. A metagenomic study using datasets and samples from multiple countries across three continents further confirmed that *Sgg* is a biomarker for CRC [9]. Adding to this strong clinical association, functional studies have demonstrated that *Sgg* plays an active role in promoting the development of colon tumors. *Sgg* stimulates the proliferation of human colon cancer cells in a manner that is dependent on β-catenin signaling [12]. *In vivo*, exposure to *Sgg* resulted in larger tumors in a xenograft model [12], and higher tumor burden and dysplasia grade in an azoxymethane (AOM)-induced CRC model [12] and in a colitis-associated CRC model [27]. The mechanism underlying the pro-tumor activity of *Sgg*, however, remains elusive.

The type VII secretion system (T7SS), also called the Esx secretion system, is a specialized secretion system first discovered in pathogenic *Mycobacterium*. It has now been found widely distributed among Firmicutes and Actinobacteria [29,30]. T7SS plays an important role in bacterial virulence and persistent infection. In *M*. *tuberculosis*, ESX-1 is by far the most prominent virulence determinant and the principal distinction between virulent mycobacteria and the attenuated BCG vaccine strains [31–38]. Consequently, T7SS proteins have been actively pursued for the development of diagnostic markers, drugs and vaccines for tuberculosis [39]. In *Staphylococcus aureus*, T7SS is important for virulence and persistent infection in a variety of disease models including kidney abscesses, nasal colonization, pneumonia, skin and blood infection [40–43].

In this study, we report the characterization of a newly identified T7SS in *Sgg* strain TX20005 (*Sgg*T7SS^T05^). We showed that *Sgg*T7SS^T05^ is active, as demonstrated by the expression of core T7SS genes *in vitro* and *in vivo*, and the secretion of a T7SS effector. Functional studies showed that *Sgg*T7SS^T05^ is important for *Sgg* adherence to CRC cells and stimulation of CRC cell proliferation. Animal studies further demonstrated that *Sgg*T7SS^T05^ is important for the colonization of the colon and is involved in the promotion of colon tumors by *Sgg*. These results reveal for the first time that T7SS is an important determinant of *Sgg* pathogenicity in the gut.

## Results

### The *Sgg*T7SS^T05^ locus

**Fig 1:**
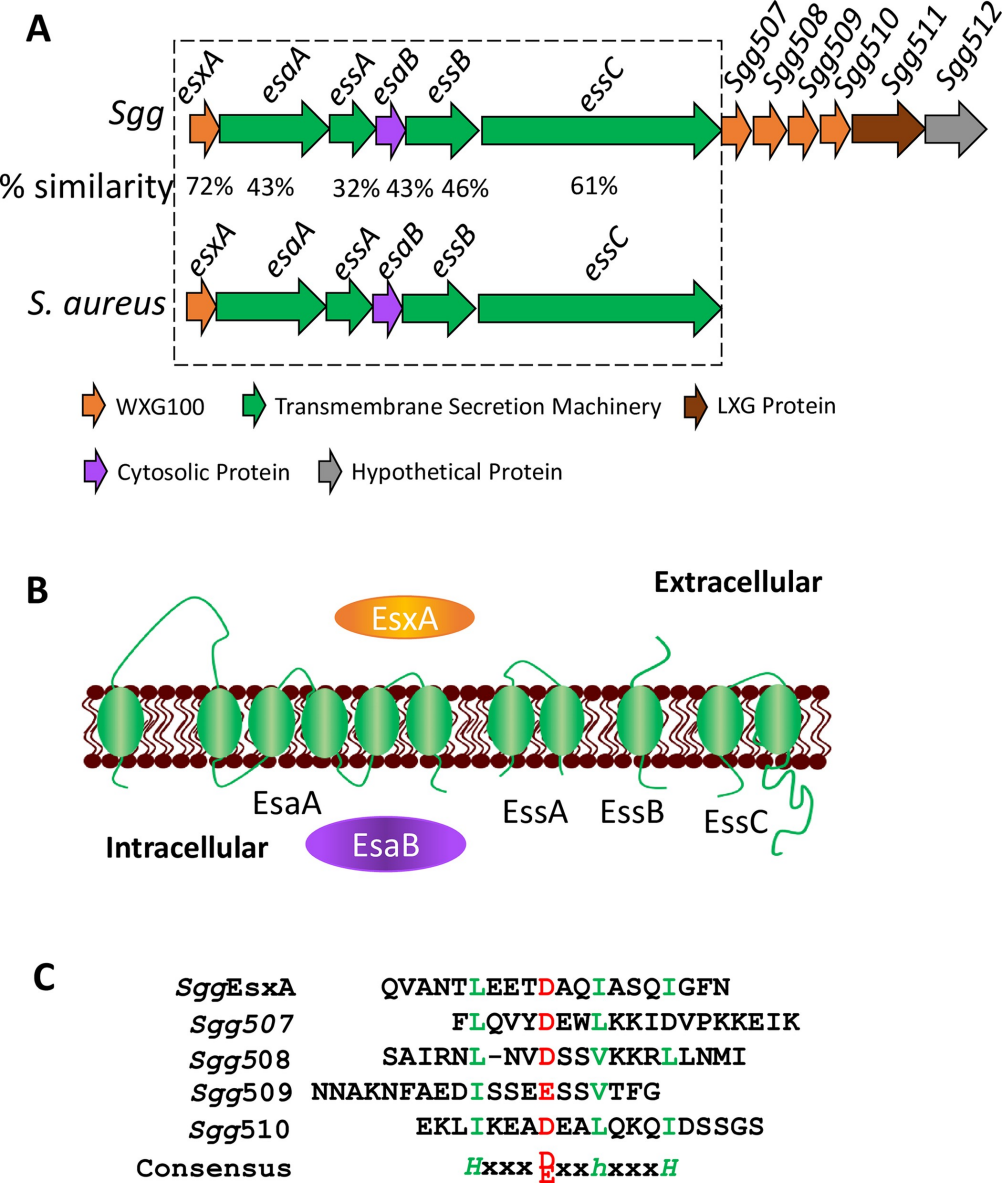
The *Sgg*T7SS^T05^ locus. **A. The genetic organization of *Sgg*T7SS**^**T05**^. The percentage beneath each gene indicates the % similarity in amino acid sequence to the corresponding protein in *S*. *aureus* USA300 [90], analyzed using Global Align at NCBI. The dashed box indicates genes encoding core components of the secretion machinery. **B. Predicted protein topology of core components of *Sgg*T7SS**^**T05**^. Membrane protein topology was predicted using Phyre2 [91]. Secretion prediction was carried out using SecretomeP 2.0a [92]. **C. The presence of a C-terminal consensus sequence motif in the 5 putative T7SS effectors within the locus.** The 20 amino acid residues at the C-terminal end of *Sgg*EsxA, and *Sgg*507 to *Sgg*510 are shown.

The genome of TX20005 was sequenced using the PacBio Sequel platform. The assembly produced one circular contig with a length of 2,258,025 bp (S1 Table). Genome annotation using the RAST [44] server identified 2,181 coding sequences, 18 rRNA operons, and 71 tRNA genes. Examination of the genome sequence revealed a putative T7SS locus that we designate as *Sgg*T7SS^T05^. All T7SSs studied so far contain two conserved features: a protein of approximately 100 amino acids with a centrally positioned WXG motif (WXG100 proteins) that is secreted by T7SS, and EssC, a transmembrane protein of the FtsK-SpoIIIE-like ATPase family that is essential for the secretion machinery. These two features are present in the *Sgg*T7SS^T05^ locus (Fig 1A). The first gene in the *Sgg*T7SS^T05^ locus encodes a protein of 97 amino acids with a central WXG motif that is highly homologous to the EsxA protein of *S*. *aureus* (49% identity and 72% similarity). Hence this gene was named *Sgg*_*esxA*. The *Sgg_esxA* gene is followed by five protein-coding genes displaying strong sequence homology to core components of the T7SS secretion machinery in *S*. *aureus* (Fig 1A). The last of these encodes a transmembrane protein highly similar to EssC of *S*. *aureus* (42% identity and 61% similarity) and is predicted to contain two FtsK/ATP-binding domains in the large C-terminal cytosolic segment. Analysis of the membrane segments and the protein topology of these putative *Sgg* T7SS core components indicates that the *Sgg* proteins also show the same topology as their respective counterparts in *S*. *aureus* (Fig 1B).

The gene immediately downstream of *Sgg_essC* (*Sgg*507) encodes a protein of 100 amino acids with ~32% similarity to EsaC, a T7SS effector of *S*. *aureus* (Burts, 2008). The remaining five genes in the locus (*Sgg*508—*Sgg*512) encode hypothetical proteins. The first three of these are 129, 93 and 100 amino acids long, each of which contains an LXG motif in the middle. Analysis by HHpred [45] predicts that each of these adopts a four-helical bundle structure typical of WXG100 proteins, with a probability of 85.3%, 97.4%, and 98.8%, respectively. The C-terminal end of WXG100 proteins is known to contain a conserved sequence motif *H*xxxD/Exx*h*xxx*H*, where *H* denotes highly conserved and *h* less conserved hydrophobic residues [46]. This motif is considered to be important for the secretion of WXG100 proteins by T7SS [46]. Examination of the *Sgg* homologs of EsxA (*Sgg*EsxA) and EsaC (*Sgg*507), as well as the three putative WXG100 proteins predicted by HHpred (*Sgg*508 –*Sgg*510) indicates that all contain this motif (Fig 1C). *Sgg*511 is a protein with 439 amino acid residues. Its N-terminal region of ~100 residues contains a central LXG motif and is predicted to fold into a four-helical bundle structure, although with a relatively low probability (36.8%). The rest of the protein does not display significant sequence homology except for a low-level similarity to bacteriocin (PF10439) at the C-terminus. This raises the possibility that this protein may belong to the LXG polymorphic toxin family [47–49]. Based on these analyses, the five genes downstream of *Sgg*_*essC* appear to encode proteins that belong in the WXG superfamily and are likely secretion substrates of T7SS [46–48,50]. The last gene in the *Sgg*T7SS^T05^ locus (*Sgg*512) is predicted to contain six transmembrane segments with unknown function.

**Fig 2:**
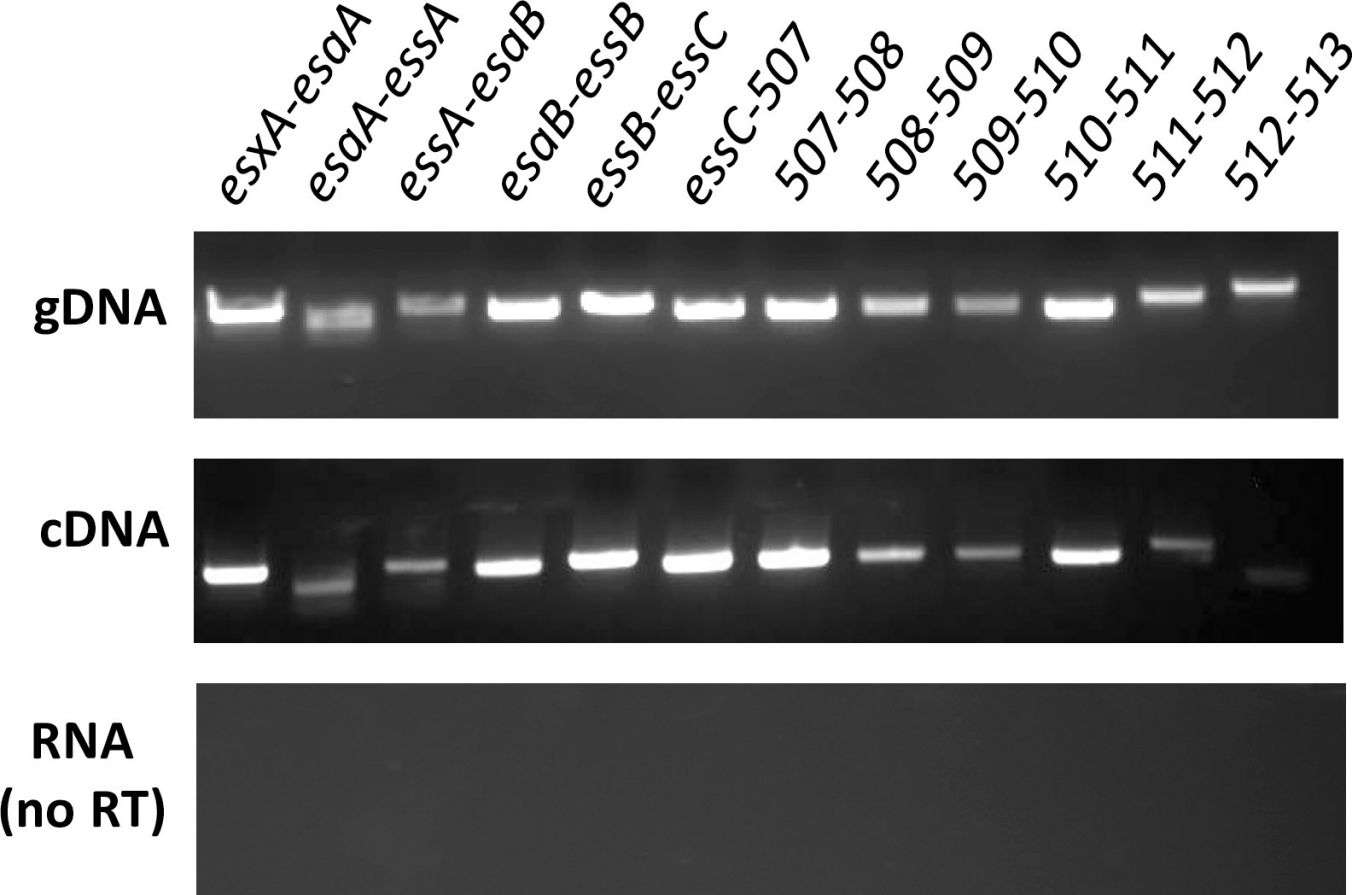
Genes in the *Sgg*T7SS^T05^ locus are transcribed in one transcriptional unit. RT-PCR was performed using primer pairs that span two adjacent genes. Genomic DNA (gDNA) from TX20005 was used as a positive control for PCR, and RNA without reverse transcriptase treatment (no RT) was used as a control for possible DNA contamination.

Genes within the *Sgg*T7SS^T05^ locus are located close to each other, with the exception of a sizeable gap of 74bp between *Sgg_esxA* and *Sgg_esaA*. Typical -35 and -10 regions were identified upstream of *Sgg_esxA* but not in the intergenic region between *Sgg_esxA* and *Sgg_esaA* using BPROM [51]. There was also no transcriptional terminator identified in this intergenic region using the iTerm-PseKNC server [52], suggesting that genes within the locus are likely transcribed as one transcriptional unit. RT-PCR was performed to further confirm this by using primer pairs that span two adjacent genes all the way across the locus. The results showed that bands of the expected size from each of the adjacent pairs can be amplified from cDNA preparations from TX20005 (Fig 2), indicating that they are indeed transcribed together. We could not PCR amplify the region spanning *Sgg*512, the last gene in the locus, and the downstream gene *Sgg*513, suggesting that *Sgg*512 is the last gene in the mRNA transcript.

### *Sgg*T7SS^T05^ encodes a functional T7SS

**Fig 3:**
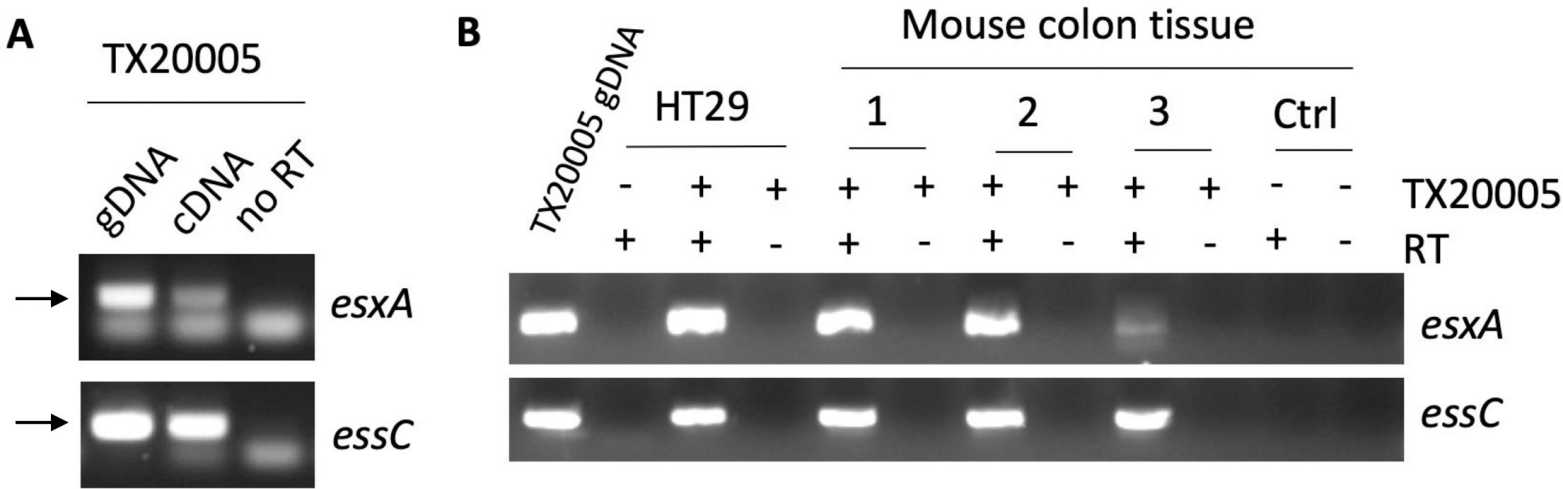
Core *Sgg*T7SS^T05^ genes are expressed *in vitro* and *in vivo*. RNA was extracted from stationary phase TX20005 grown in BHI (**A**), co-cultured with HT29 (**B**), or from colonic tissues from mice gavaged with TX20005 (**B**) and analyzed by RT-PCR. RNA samples untreated with the reverse transcriptase (no RT) was used a control for DNA contamination. Genomic DNA from TX20005 (gDNA) was used as a control for the PCR reactions.

We next sought to determine if core genes within the *Sgg*T7SS^T05^ locus are expressed. We focused on the two conserved genes of T7SS, *Sgg*_*esxA* and *Sgg*_*essC*. Expression of these genes was analyzed by RT-PCR. We found that both *Sgg*_*esxA* and *Sgg*_*essC* were expressed in TX20005 grown in brain heart infusion (BHI) broth (Fig 3A, indicated by arrows) and when TX20005 was co-cultured with the CRC cell line HT29 (Fig 3B). To determine if these genes are expressed *in vivo*, we collected colon tissues from mice orally gavaged with TX20005 or saline and extracted tissue RNA. We found that both *Sgg*_*esxA* and *Sgg*_*essC* were expressed in the colonic tissues. We did not detect any PCR amplification products in saline-treated mice, indicating the specificity of the PCR method. In addition, bands of the expected size were not observed in PCR reactions using RNA preparations that had not been treated with reverse transcriptase, indicating that there is minimal DNA contamination in the RNA samples. Taken together, these results indicate that *Sgg*_*esxA* and *Sgg*_*essC* are expressed *in vitro* and *in vivo*.

**Fig 4:**
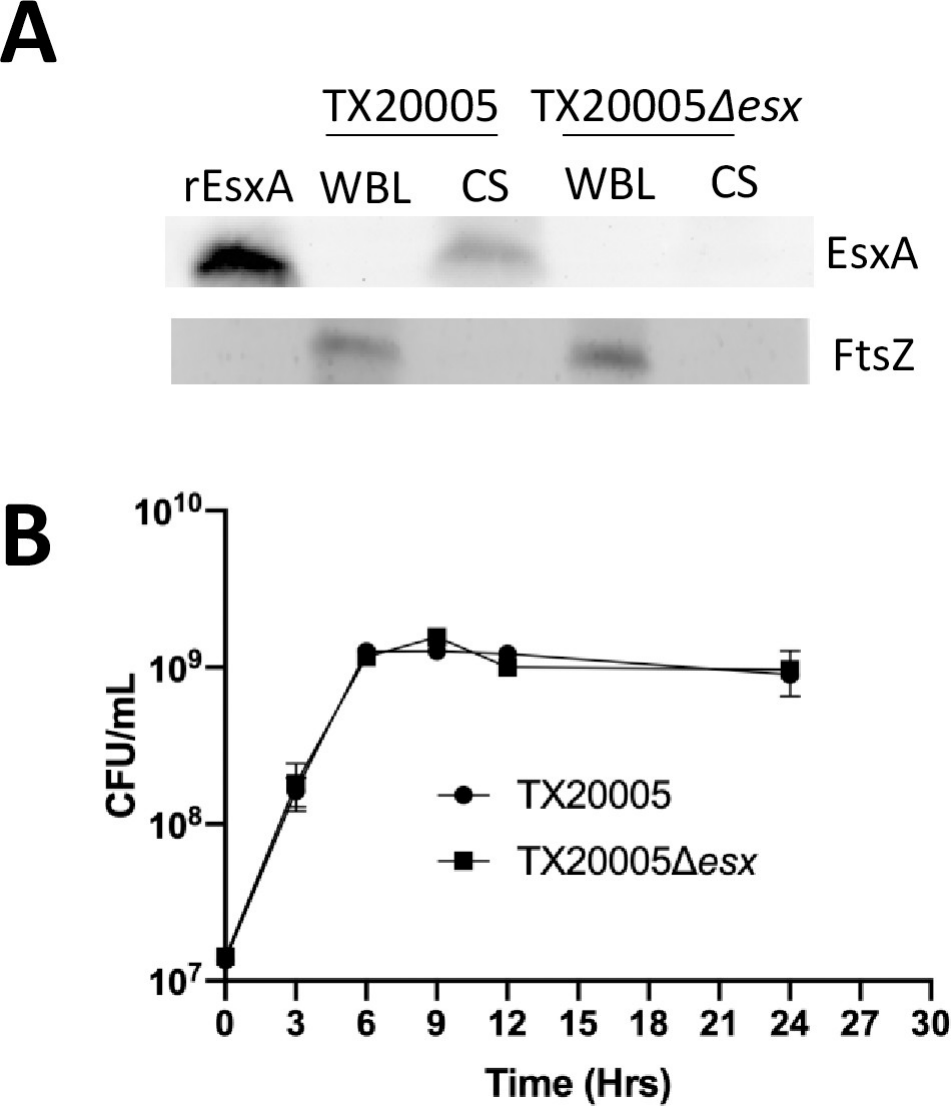
*Sgg*T7SS^T05^ is functional. **A.** *Sgg*EsxA is secreted. TX20005 and TX20005Δ*esx* were grown in BHI broth with shaking for ~ 18 hours. Whole bacterial lysates (WBL) and culture supernatants (CS) were analyzed by western blot, as described in the Materials and Methods section. An equivalent of ~ 0.5 ml of overnight cultures was loaded onto an SDS gel. FtsZ was used as a cytosolic protein control. Purified tag-free recombinant *Sgg*EsxA protein (rEsxA) was used as a positive control for the protein. **B.** Growth curves of TX20005 and TX20005*Δesx*. Data shown are the mean ± SEM and combined from three independent experiments.

We next sought to examine the secretion activity of *Sgg*T7SS^T05^. Tag-free recombinant *Sgg*EsxA protein (rEsxA) (S1 Fig) was obtained and used to generate rabbit antiserum. The anti-serum was used to determine if *Sgg*EsxA was secreted from TX20005 by western blot. We were able to detect a band with the expected molecular weight for *Sgg*EsxA (~ 10.9 kDa) in the culture supernatants (CS) from TX20005 but not in whole bacterial lysates (WBL) (Fig 4A). We generated a deletion mutant in which the DNA region encoding the core secretion machinery (*Sgg_esxA* to an N-terminal portion of *Sgg_essC*) was deleted. This resulted in a mutant (TX20005*Δesx*) missing the entire secretion machinery. TX20005*Δesx* did not have any growth defects compared to the parent strain TX20005 when grown in BHI broth (Fig 4B). The deletion also did not affect the transcription of the upstream (*Sgg*500) or the downstream (*Sgg*507) genes flanking the deleted region (S2 Fig). As expected, we did not detect a band of the expected molecular weight for *Sgg*EsxA in either the CS or WBL from the deletion mutant (Fig 4A and S3 Fig), indicating that the band we observed in the CS of TX20005 is specific for *Sgg*EsxA. An antibody against the cell division protein FtsZ was used as a cytosolic loading control. The antibody detected a band with the expected molecular weight in the WBL from both TX20005 and the deletion mutant, but not in the CS from either strain, suggesting that the EsxA band we observed in the CS from TX20005 is secreted rather than released from bacterial lysis. Taken together, these results indicate that the *Sgg*T7SS^T05^ locus encodes a functional T7SS.

### *Sgg*T7SS^T05^ is required for adherence of *Sgg* to CRC cells

**Fig 5:**
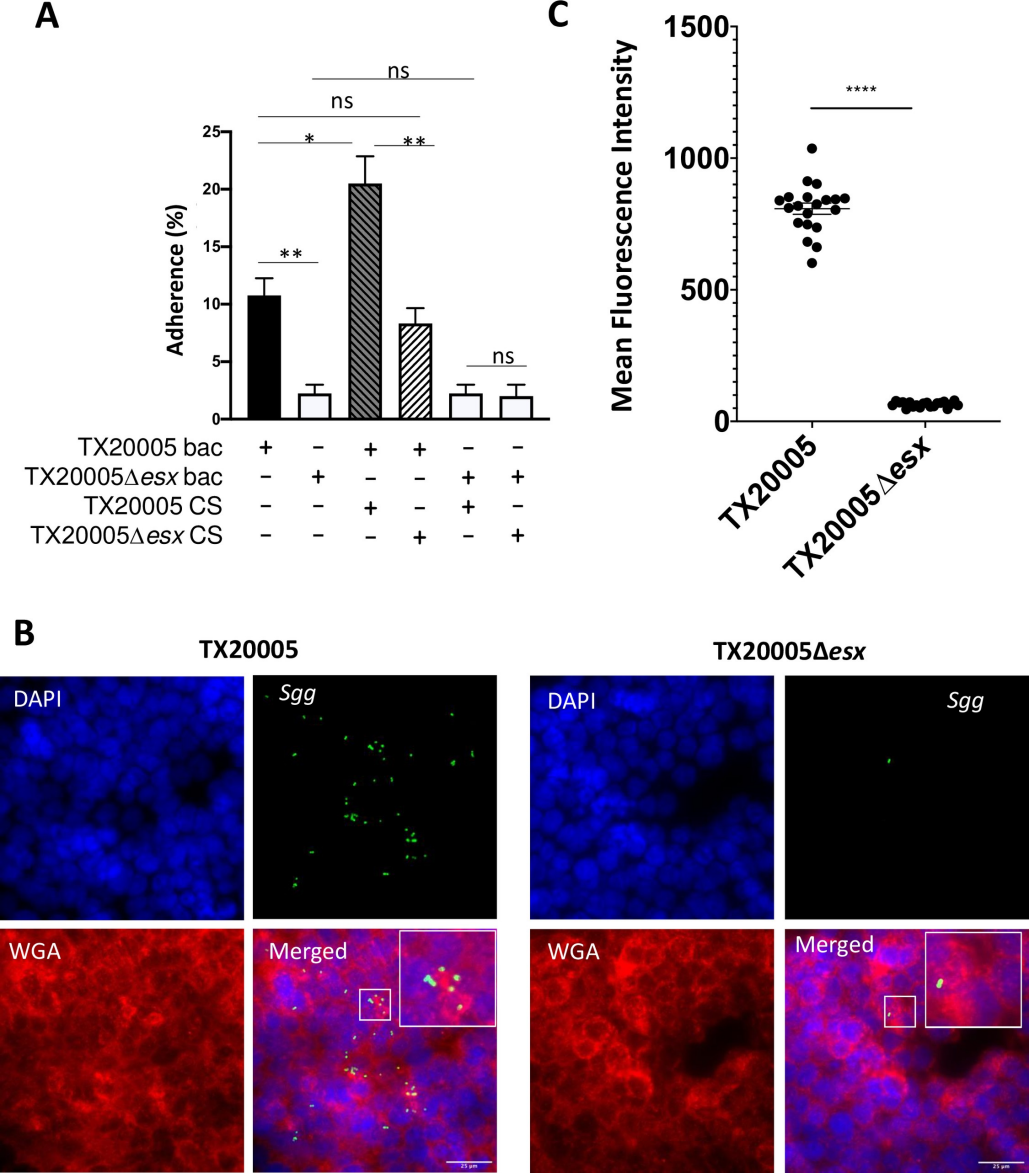
Disruption of *Sgg*T7SS^T05^ impairs *Sgg* adherence to host cells. **A.** HT29 cells were incubated with TX20005 or TX20005Δ*esx* (MOI = 10), supplemented with or without CS from TX20005 or TX20005Δ*esx* for 1hr. The cells were washed and attached bacteria were enumerated by dilution plating, as described in the Materials and Methods section. Adherence was calculated as the percentage of adhered bacteria vs. total bacteria added. Data shown are the mean ± SEM and combined from three independent experiments. Ns, not significant; *; *p* < 0.05; **, *p* < 0.01; unpaired two-tailed *t* test. **B.** Immunofluorescence microscopy was performed as described in the Materials and Methods section. Representative images are shown. **C**. Fluorescence intensity at 488 nm was quantified using Image J on 20 randomly selected fields for each group. ****, *p* < 0.0001, unpaired two-tailed *t* test.

Interactions with colonic epithelial cells are important for *Sgg* pathogenicity in the gut. Previous studies showed that *Sgg* is capable of adhering to human CRC cell lines [12,53]. We sought to determine if *Sgg*T7SS^T05^ is important for *Sgg* adherence to host cells. The results showed that TX20005*Δesx* adheres to HT29 cells at a significantly reduced level compared to that of TX20005 (~68% reduction) (Fig 5A). Immunofluorescence microscopy was also performed. The wild type and the mutant bacteria display similar morphology, appearing as single cells or in short chains as expected (Fig 5B). Again, the mutant adhered to HT29 at a significantly reduced level compared to TX20005 (Fig 5B and 5C), further confirming the result from using the plating method.

We next examined if *Sgg*T7SS^T05^-secreted factors are involved in *Sgg* adherence to host cells. CS from TX20005 and TX20005*Δesx* were filter-sterilized and added to the WT or the mutant bacteria in the adherence assay. The results showed that the adherence of TX20005 to HT29 cells was significantly enhanced by WT CS, whereas CS from the mutant had no effect. Interestingly, the CS from neither TX20005 nor the mutant had any effect on the adherence of the mutant bacteria (Fig 5A), indicating that WT CS alone is insufficient to restore the adherence of TX20005*Δesx* to the WT level and that bacterial surface associated molecules are also required. Taken together, these results suggest that *Sgg*T7SS^T05^-secreted factors likely act as an adaptor that bridges *Sgg* surface associated molecules with a host cell receptor(s). Furthermore, the results suggest that the bacterial surface molecules involved in adherence are also dependent on a functional T7SS.

### *Sgg*T7SS^T05^ is required for *Sgg-*induced CRC cell proliferation

**Fig 6:**
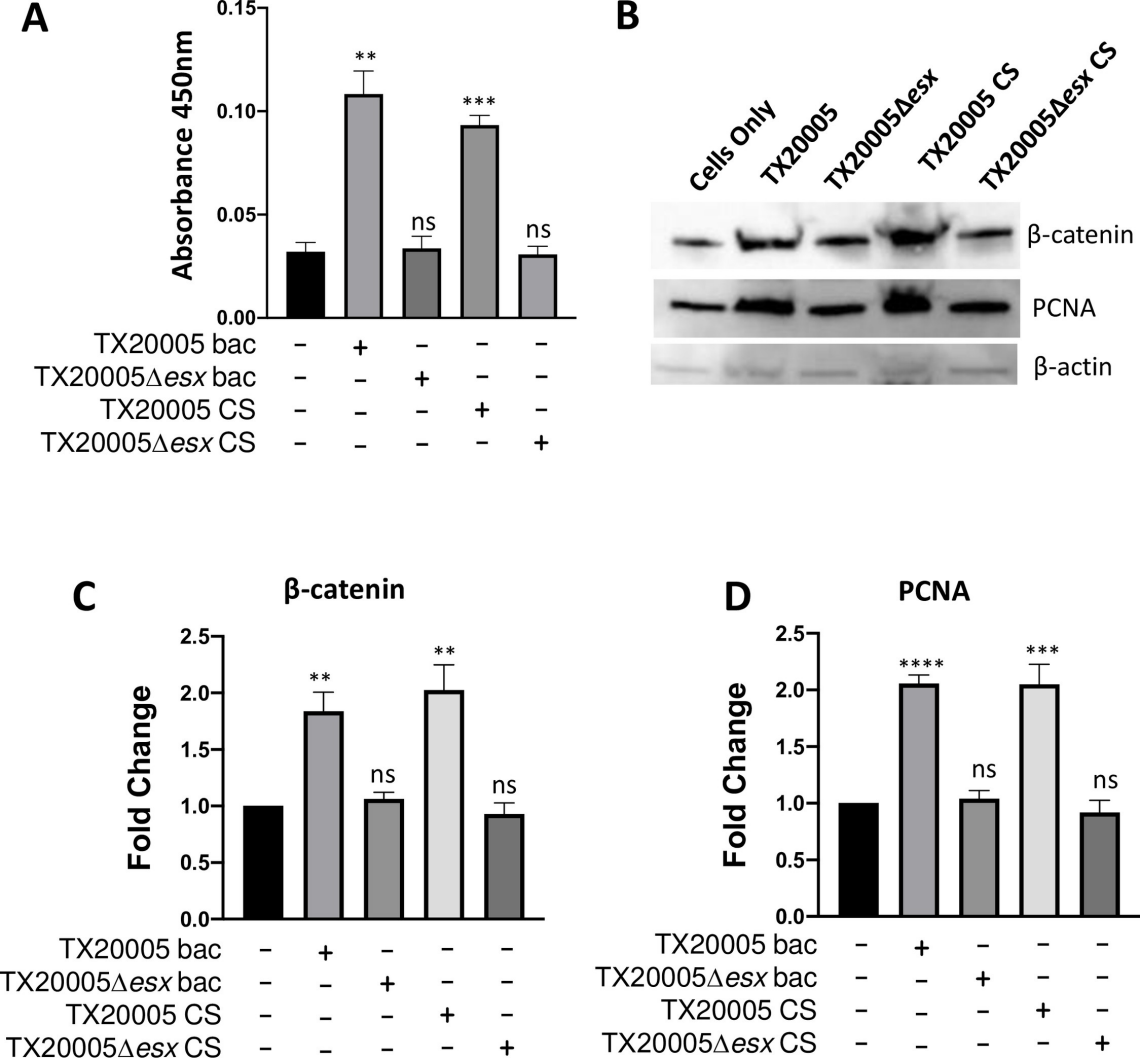
*Sgg*T7SS^T05^ is required for *Sgg* to stimulate host cell proliferation. **A. Cell proliferation assay.** HT29 cells were incubated with TX20005 or TX20005Δ*esx* bacteria (MOI = 1), or in media supplemented with CS from TX20005 or TX20005Δ*esx* for 24 hours. Viable cells were quantified using the CCK-8 assay. **B-D. Western blot.** HT29 cells were incubated with TX20005 or TX20005Δ*esx* bacteria (MOI = 1), or in media supplemented with CS from TX20005 or TX20005Δ*esx* for 9 hours. Whole cell lysates were analyzed by western blot, probed with antibodies against β-catenin, PCNA and β-actin. Representative images are shown (**B**). Band intensity was quantified using ImageJ, normalized to β-actin first, and then to cells only to calculate fold changes. Results shown are mean ± SEM, combined from at least three independent experiments (**C** and **D**). ns, not significant; *; *p* < 0.05; **, *p* < 0.01; ***, *p* < 0.001; ****, *p* < 0.0001; unpaired two-tailed *t* test, vs. cells only.

TX20005 is able to directly stimulate the proliferation of CRC cells in a β-catenin-dependent manner [12]. We tested the effect of disrupting *Sgg*T7SS^T05^ on the pro-proliferative capacity of *Sgg*. HT29 cells were cultured in the presence or absence of WT and mutant bacteria for 24 hours. TX20005 significantly increased cell proliferation as expected, whereas deletion of T7SS abrogated the ability of TX20005 to stimulate cell proliferation (Fig 6A). We confirmed that there is no difference in bacterial titers between TX20005 and TX20005*Δesx* during these cell proliferation assays (S4 Fig). We next tested the effect of CS on cell proliferation by incubating HT29 cells in media supplemented with filter-sterilized CS from TX20005 or TX20005*Δesx*. CS from TX20005 significantly increased cell proliferation compared to cells cultured in media only, whereas CS from TX20005*Δesx* had no effect (Fig 6A). To further validate this finding, we performed western blot analysis to probe for β-catenin and proliferating cell nuclear antigen (PCNA). The results showed that both β-catenin and PCNA were significantly increased in cells co-cultured with TX20005 bacteria or CS, whereas no increase was observed in either proteins in cells cultured with the mutant bacteria or CS (Fig 6B–6D). Taken together, these results indicate that *Sgg*T7SS^T05^-secreted factors are able to directly stimulate host cell proliferation.

### *Sgg*T7SS^T05^ is important for the colonization of colon by *Sgg*

**Fig 7:**
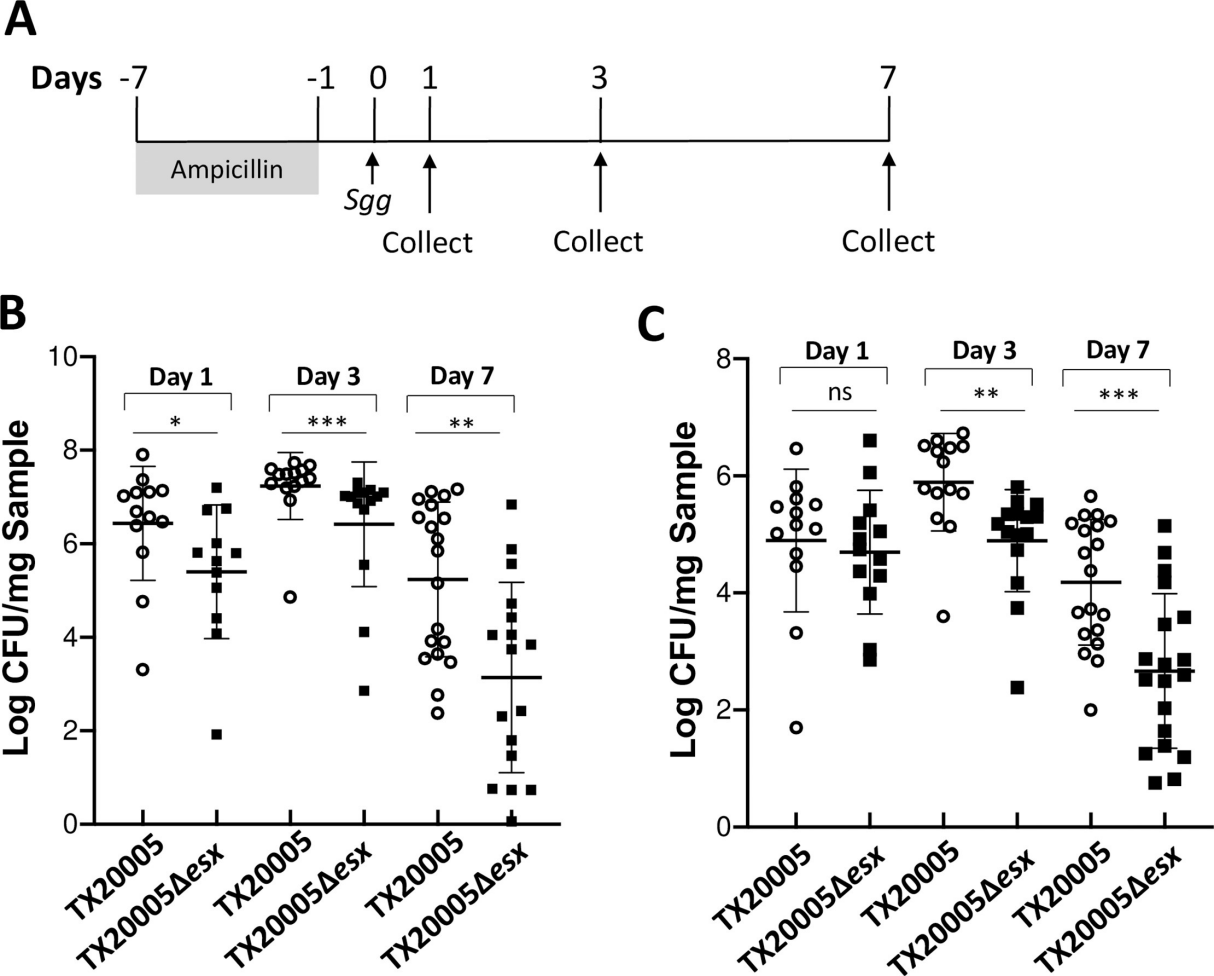
Deletion of *Sgg*T7SS^T05^ reduces the colonization capacity of TX20005. The ability of TX20005 and TX20005Δ*esx* to colonize the mouse colon was examined in a mouse colonization model, as shown in **A**. Fecal materials (**B**) and colonic tissues (**C**) were homogenized and dilution plated onto Enterococcus Selective Agar plates to enumerate *Sgg*. Results were normalized to per mg sample and combined from two independent experiments (n = 12–19 mice/group). Error bars indicate the standard deviation. ns, not significant; *; *p* < 0.05; **, *p* < 0.01; ***, *p* < 0.001; Mann-Whitney test.

We investigated if *Sgg*T7SS^T05^ is important for the colonization of the colon by *Sgg* in a mouse colonization model (Fig 7A) [26]. Fecal materials and colon tissues were collected at day 1, 3, and 7 post bacterial oral gavage, weighed, homogenized, and plated onto selective agar plates to enumerate live *Sgg*. We found that the *Sgg* load in the fecal materials from mice gavaged with TX20005*Δesx* was significantly lower than that in the TX20005 group at all three time points (Fig 7B). In the colonic tissues, the *Sgg* load from mice gavaged with the mutant was similar to that in mice gavaged with TX20005 at day 1, but significantly decreased at day 3 and 7 (Fig 7C). We further investigated the ability of WT and mutant *Sgg* to colonize the distal and proximal portion of the colon. We found no difference in the *Sgg* load between these two sites for either TX20005 or the mutant at day 3 or 7 (S5A and S5B Fig), suggesting that *Sgg*T7SS^T05^ contributes to *Sgg* colonization of both the distal and proximal colon.

Taken together, the results described here indicate that *Sgg*T7SS^T05^ is important for the colonization of the colon by *Sgg*.

### *Sgg*T7SS^T05^ is important for the promotion of colon tumors

**Fig 8:**
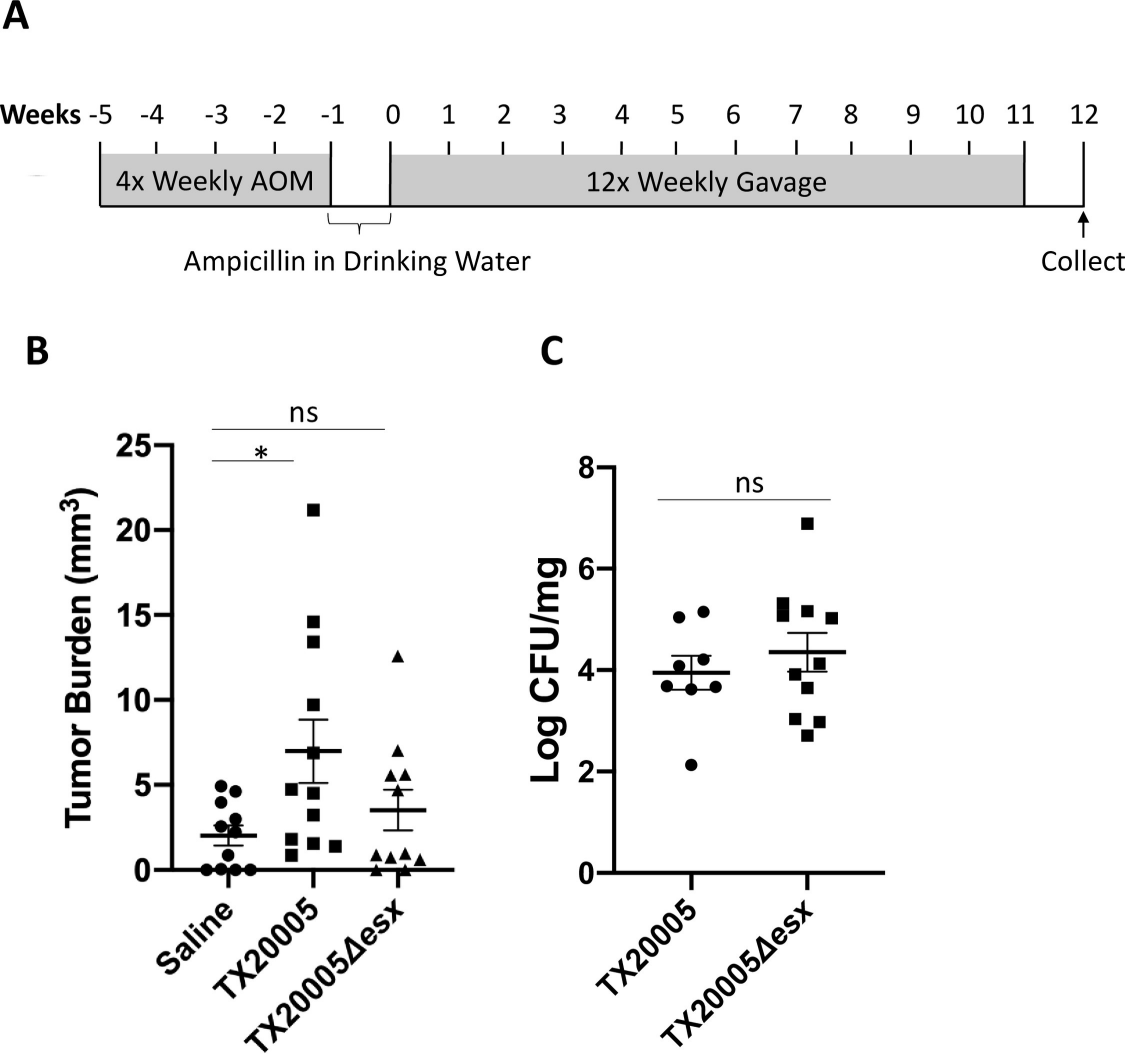
Deletion of *Sgg*T7SS^T05^ impairs the ability of *Sgg* to promote the development of colon tumors. The effect of TX20005 and TX20005Δ*esx* on the development of colon tumors was examined in an AOM-induced mouse model of CRC, as shown in **A**. Macroscopic tumors were evaluated by blinded observers. Tumor burden (**B**) was calculated as the sum of tumor volumes from one mouse. Fecal materials were homogenized and dilution plated onto Enterococcus Selective Agar plates to enumerate *Sgg* bacteria (**C**). Results were combined from two independent experiments (n = 11–12 mice/group). The mean ± SEM are indicated in the graph. *, *p* < 0.05; ns, not significant; Mann-Whitney test.

We next investigated if *Sgg*T7SS^T05^ plays a role in the development of colon tumors in an AOM-induced mouse model of CRC (Fig 8A). As expected, mice treated with WT TX20005 exhibited significantly increased tumor burden compared to mice treated with saline alone (Fig 8B). However, mice exposed to TX20005*Δesx* did not show a significant increase in the tumor burden compared to the saline group, suggesting that the mutant is attenuated in promoting the development of colon tumors. In addition, the mean tumor burden in TX20005Δ*esx*-treated group is lower than that in TX20005-treated group, although the difference is not statistically significant (*p* = 0.1010, Mann-Whitney test). It is likely that *Sgg*T7SS^T05^ is not the only factor important for the promotion of colon tumors by *Sgg*, other factors are also involved. Interestingly, we did not observe any difference in the *Sgg* burden between TX20005-treated and the mutant-treated groups (Fig 8C). This could be due to the repeated oral gavage of bacteria during the animal procedure, which could have masked the difference in the colonization capacity between the WT and mutant strains, or that *Sgg*T7SS^T05^ is not involved in colonizing tumor-bearing colons. These results, combined with the pro-proliferative effect of CS from TX20005 shown in Fig 5, suggest that *Sgg*T7SS^T05^-secreted factors are directly involved in promoting the development of colon tumors.

## Discussion

*Sgg* has a long-standing clinical association with CRC [18–25,54–67]. Recent functional studies using pre-clinical models of CRC further demonstrate that *Sgg* actively promotes the development of colon tumors [12,27]. The molecular determinants responsible for *Sgg* pathogenicity in the colon, however, were unknown. Here we report the characterization of a newly identified T7SS of *Sgg* and show that this T7SS is an important player in; colonization of the colon, interactions with CRC cells, and promotion of colon tumors. To our knowledge, this is the first characterization of T7SS in *Sgg*, the first direct evidence that T7SS is a critical determinant for *Sgg* pathogenicity in the colon, and the first identification of a molecular pathway important for the pro-tumor activity of *Sgg*.

We found that *Sgg*T7SS^T05^ displays a strong similarity to *S*. *aureus* T7SS in both genetic organization and amino acid sequence of the core components. Further analysis of the proteins encoded by genes within this locus identified six putative secretion substrates of *Sgg*T7SS^T05^: *Sgg*EsxA, and *Sgg*507 to *Sgg*511. The first five of these proteins display features indicative of WXG100 proteins: ~100 amino acid residues in length, a centrally positioned W/LXG motif, a predicted four-helix bundle structure typical of WXG100 proteins, and a C-terminal conserved sequence motif *H*xxxD/Exx*h*xxx*H* [46,50]. The last of the six proteins (*Sgg*511) shows an N-terminal similarity to the WXG100 proteins and a low-level similarity to bacteriocin, suggesting that it may belong to the LXG polymorphic toxin family [47–49].

The ability of *Sgg* to interact with the colonic epithelium and influence epithelial homeostasis and function is key to its pathogenicity in the colon. *Sgg* was previously shown to adhere to colonic epithelial cell lines but not invade these cells [12,53,68]. The Pil3 pilus of *Sgg* is able to bind to mucin and fibrinogen [69] and mediates *Sgg* adherence to HT29-MTX, a high mucin producer, but not to HT29 [68]. Our results indicate that *Sgg*T7SS^T05^ is important in *Sgg* adherence to HT29 cells, suggesting that *Sgg*T7SS^T05^-mediated adherence likely involves a novel mechanism distinct from that of Pil3. The fact that CS from TX20005 enhances the adherence of TX20005 but does not increase the adherence of TX20005Δ*esx* further suggests that *Sgg*T7SS^T05^-mediated adherence involves both secreted and bacterial surface associated factors. It is possible that *Sgg*T7SS^T05^-secreted factors act as adaptors linking bacterial surface associated proteins to host cell receptors, as proposed previously from structural analysis of WXG100 proteins [70]. The inability of TX20005 CS to restore the adherence capacity of the mutant suggests that the relevant bacterial surface associated factors also depend on a functional T7SS. This can happen in a number of ways. Recent work in *S*. *aureus* suggests that T7SS secretes or stabilizes peripheral membrane proteins [71]. Work from Lou *et al*. suggests that there may be a T7SS secretion needle [72,73]. In addition, disruption of Esx-5 in mycobacteria affected bacterial surface hydrophobicity, which can influence bacterial interaction with host cells or the association of bacterial proteins with the surface [74].

*Sgg* was previously shown to stimulate the proliferation of colon cancer cells *in vitro* and *in vivo* and this effect requires β-catenin [12]. The *Sgg* molecules responsible for this pro-proliferative effect were unknown. Here we demonstrate that disruption of T7SS abolishes the ability of *Sgg* to stimulate cell proliferation, or to upregulate β-catenin or PCNA. Thus, *Sgg*T7SS^T05^ is required for *Sgg* to stimulate host cell proliferation. The fact that TX20005 CS alone stimulated host cell proliferation whereas CS from the mutant did not suggest that *Sgg* stimulation of host cell proliferation is mediated by *Sgg*T7SS^T05^-secreted proteins. This is consistent with a recent study showing that CS from several *Sgg* strains were able to directly promote HT29 cell proliferation in the absence of bacteria [75]. At the moment, it is unclear whether the *Sgg*T7SS^T05^-secreted factor mediating cell proliferation is the same as the one that enhances *Sgg* adherence to host cells. We previously showed that *Sgg* strains that adhered poorly were also unable to stimulate cell proliferation [26]. It is possible that the secreted factor responsible for stimulating cell proliferation is the same as the one that bridges the bacteria to the host cells. However, the possibility that distinct factors are involved in adherence and proliferation cannot be excluded. Further investigations to identify the specific secreted factors involved in these processes are needed.

**Fig 9:**
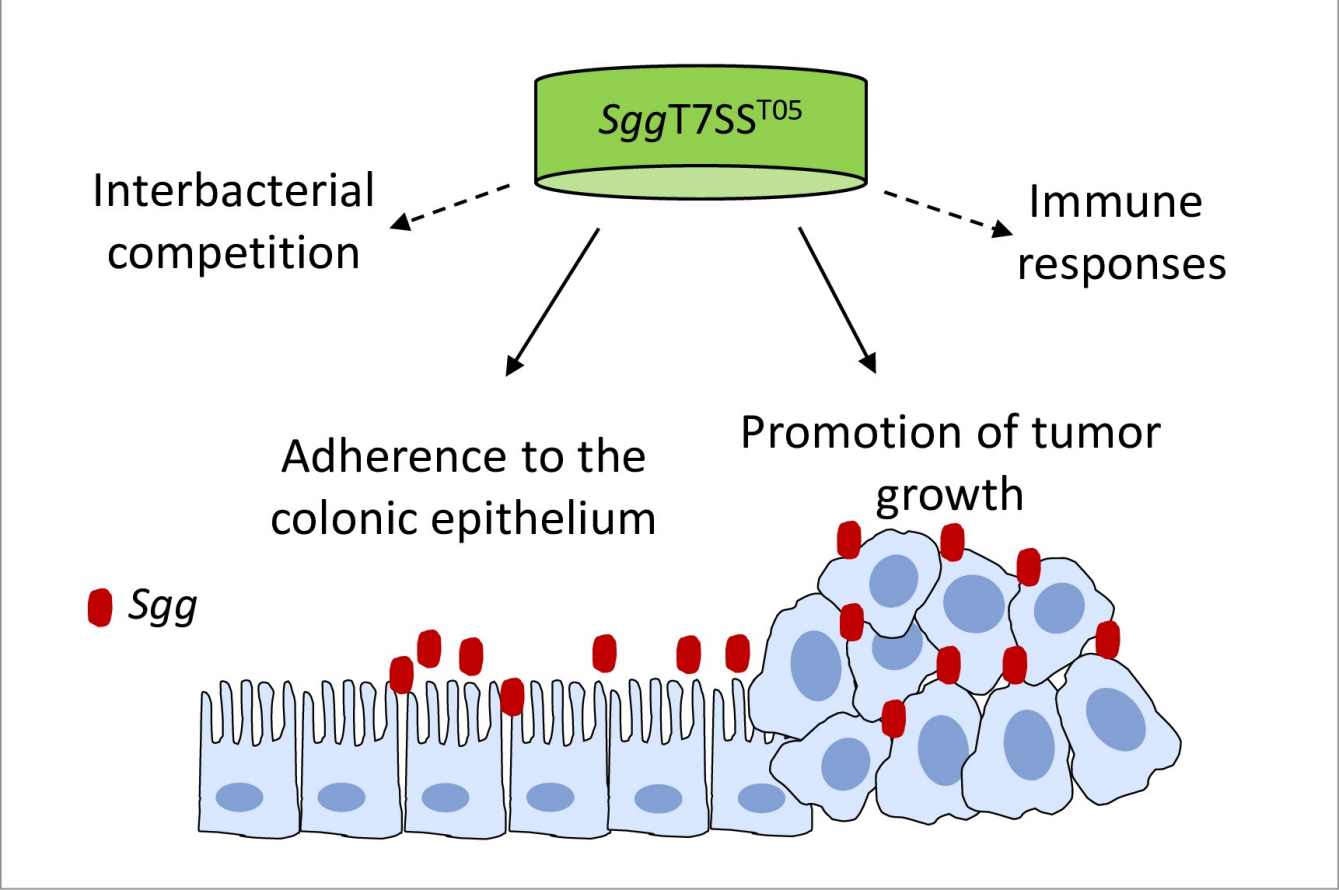
A working model for how *Sgg*T7SS^T05^ contributes to *Sgg* pathogenicity in the colon.

Colonization of the colon by *Sgg* affords *Sgg* the opportunity to exert its influence in the colon. Our data indicates that *Sgg*T7SS^T05^ is important for *Sgg* to colonize the colon. Deletion of *Sgg*T7SS^T05^ significantly reduced *Sgg* load in the fecal materials and the colonic tissues. Previous work indicated that gallocin, a bacteriocin produced by *Sgg*, mediates *Sgg* colonization of the proximal portion of tumor-bearing colons [76]. Here we showed that *Sgg*T7SS^T05^ is involved in *Sgg* colonization of both the proximal and distal portion of normal colons. Thus, *Sgg*T7SS^T05^-mediated *Sgg* colonization of the colon represents a novel colonization mechanism for *Sgg*. There are a number of ways *Sgg*T7SS^T05^ mediates gut colonization (Fig 9). The reduced *Sgg* load in the colonic tissues could be due to the impaired ability of *Sgg* to adhere to the colonic epithelium, as we showed that disruption of *Sgg*T7SS^T05^ impaired *Sgg* adherence to host cells. In addition, LXG toxins secreted by T7SS mediate interbacterial antagonism [49,77]. Sequence analysis of the *Sgg*T7SS^T05^ locus suggests that one of the genes (*Sgg511*) may encode a potential LXG toxin, thereby providing a competitive advantage and contributing to its survival in the gut. Lastly, T7SS is known to modulate host immune responses. It is important for the escape of mycobacteria from macrophage phagosomes [78–81] and the interaction with the cytosolic DNA sensor nucleotidyltransferase cyclic GMP–AMP synthase (cGAS) [82–84]. Effectors secreted by the T7SS of *S*. *aureus* stimulate IL-12 signaling [85] and control dendritic cell function [86]. *Sgg* is reported to induce subdued immune responses. The cytokine profile in the mouse colon following exposure to *Sgg* is similar to that following exposure to a common gut commensal *Lactococcus lactis* [12]. *Sgg* also selectively recruits bone-marrow derived suppressor cells and inhibits CD4^+^ T cells, resulting in an immune tolerant microenvironment [27]. Transcriptomic analysis revealed that *Sgg* induces lower cytokine expression in macrophages compared to *S*. *aureus* [87]. Whether *Sgg*T7SS^T05^ plays a role in this subdued immune response is currently unknown. Further investigation is needed to gain insight into the involvement of *Sgg*T7SS^T05^ in regulating host immune responses.

Results from experimental models indicate that *Sgg* actively promotes the development of colon tumors [12,27], however, the *Sgg* molecules that mediate the tumor-promotion activity were unknown. Our results from the AOM-induced CRC model indicate that disruption of *Sgg*T7SS^T05^ impaired the ability of *Sgg* to promote colon tumors. This combined with the lack of difference in the *Sgg* load between the WT and mutant bacteria-treated mice and the observed effect of TX20005 CS on cell proliferation suggest that *Sgg*T7SS^T05^ contributes to the development of colon tumors through, at least in part, a direct effect of *Sgg*T7SS^T05^-secreted factors on tumor cell proliferation. Our results also suggest that *Sgg* factors other than *Sgg*T7SS^T05^ are likely to be involved in tumor promotion as well. This is not surprising as previous studies suggest that *Sgg* promotes the development of colon tumors via multiple mechanisms. *Sgg* was shown to upregulate β-catenin signaling and the upregulation is essential for *Sgg* to stimulate cell proliferation and tumor growth [12]. *Sgg* also induces an immune tolerant microenvironment that favors tumor progression [27]. Further investigation into the biological activities mediated by specific *Sgg*T7SS^T05^ effectors will be important to dissect the molecular mechanism underlying the pro-tumor capability of *Sgg*.

In summary, we report the first characterization of a T7SS in *Sgg* and demonstrate that it plays an important role in *Sgg* interactions with colonic epithelial cells, colonization of the colon and promotion of colon tumors. Based on the aforementioned results and discussion, a working model for how *Sgg*T7SS^T05^ mediates these activities is proposed (Fig 9). Antibacterial toxins secreted by *Sgg*T7SS^T05^ may inhibit the growth of other commensal bacteria in the gut, thereby affording a competitive growth advantage to *Sgg*. Adherence to the colonic epithelium further facilitates *Sgg* colonization of the colonic tissues. Close proximity to the colonic epithelium allows effectors secreted by *Sgg*T7SS^T05^ to target host cell receptors and induce signaling activities involved in cell proliferation. Lastly, effectors secreted by *Sgg*T7SS^T05^ may also modulate immune responses in the gut, creating an immune suppressive environment favorable for *Sgg* survival and tumor development. Further investigation into the activities of specific *Sgg*T7SS^T05^ effectors will be important to elucidate the molecular pathways organized by *Sgg*T7SS^T05^ that regulate keys aspects of *Sgg* pathogenicity in the gut. These specific effectors are likely candidates for biomarkers with strain-level resolution, and targets for clinical intervention.

## Materials and methods

### Bacterial strains, cell lines and growth conditions

*Sgg* strains [12] were grown in brain heart infusion (BHI) agar or broth (Teknova). For co-culture experiments and animal studies, stationary phase bacterial cultures were pelleted, washed with PBS, resuspended in phosphate-buffered saline (PBS) containing 15% glycerol, aliquoted and stored at -80°C. Stocks were washed with PBS, and properly diluted to obtain the required concentration. Human colon cancer cell line HT29 (ATCC) was grown in Dulbecco’s Modified Eagle’s Medium F-12 50/50 (DMEM/F-12, GIBCO) supplemented with 10% FBS at 37°C with 5% CO_2_ in a humidified chamber. Cells between passages 20 and 30 were used in the experiments.

### Genome Sequencing of TX20005

Genome sequencing of the *Sgg* strain TX20005 was performed using the PacBio Sequel platform (Pacific Biosciences). Read correction and *de novo* assembly was completed using Canu version 1.7.1 [88]. Assembly produced one circular contig with a length of 2,258,025 bp and nearly 174X depth of coverage. Genome polishing was conducted using Arrow (https://github.com/PacificBiosciences/GenomicConsensus; Pacific Biosciences), a tool that builds a final consensus sequence by mapping raw data against the assembled contig. The resulting sequence was subsequently annotated via the Rapid Annotations using Subsystems Technology (RAST) [44] server sponsored by the National Microbial Pathogen Database Resource (https://rast.nmpdr.org). Raw and corrected read metrics are shown in S1 Table.

### Adherence assay

**1) Plate assay**. This was performed as described previously [12]. Briefly, HT29 cells were seeded at a density of 1x10^6^ cells/well in a 24 well plate and allowed to attach overnight. Cells were incubated with bacteria at a multiplicity of infection (MOI) of 10 for 1 hour at 37°C with 5% CO_2_. Cells were then washed with PBS, lysed with PBS containing 0.025% Triton-X 100 and dilution plated. Adherence was calculated as the percentage of adherent bacteria vs. total bacteria added. **2) Immunofluorescence microscopy.** HT29 cells were seeded onto Nunc Lab-Tek II Chamber slides at a density of 5x10^5^ cells per well. Bacteria were added to the cells (MOI = 10) and incubated for 1 hour. Cells were washed with PBS, fixed in 100% ice-cold methanol for 10 minutes at -20°C, and washed again with PBS. Cells were blocked in blocking buffer (PBS containing 1 mg/ml saponin and 2% (w/v) bovine serum albumin) for 30 minutes at room temperature, and then incubated with rabbit anti-*Sgg* (1:250) [12] and Alexa Fluor™ 594 conjugated wheat germ agglutinin (1:250) (Thermo Fisher) overnight at 4°C in the dark, followed by incubation with goat anti-rabbit IgG Alexa Fluor 488 Conjugate and counterstained with DAPI (1:2500). Slides were viewed in a DeltaVision confocal microscope. Fluorescence intensity at 488nm was quantified on 20 randomly selected fields for each group using ImageJ.

### Cell proliferation assay

This was performed as described previously [12] with modifications. Briefly, HT29 cells were seeded in 96 well plates at a concentration of ~1x10^4^ or ~5x10^4^ cells/well and incubated overnight to allow cells to attach. Cells were then incubated in fresh DMEM/F-12 with 10% FBS containing *Sgg* bacteria (MOI = 1) for a total of 24 hours. Trimethoprim (1 μg/mL final concentration) was added after 6 hours of incubation to inhibit bacterial growth, as previously described [12]. The number of viable cells was determined using the CCK-8 kit following the instructions of the supplier (Apex Bio).

### Preparation of *Sgg* culture supernatants

Overnight cultures of *Sgg* grown in BHI broth were centrifuged. The supernatants were filtered through a 0.2 μm filter and then concentrated ~ 10-fold using Pierce™ Protein Concentrator PES, 3K Molecular Weight Cutoff, aliquoted and stored at -80°C. For adherence and cell proliferation assays, concentrated supernatants were reconstituted in the appropriate tissue culture media to 1X.

### RNA extraction, cDNA synthesis and PCR

**1) From colonic tissues.** Colonic tissues were collected one day after mice were orally gavaged with *Sgg* strain TX20005 (~1x10^8^ CFU/mouse) or saline. RNA was extracted using the DNA/RNA/Protein Allprep kit (Qiagen). **2) From co-cultures with HT29 cells**. RNA was extracted from HT29 cells following 24-hour incubation with or without bacteria (MOI = 1) using the DNA/RNA/Protein Allprep kit (Qiagen). **3) From *Sgg* cultures**. RNA was extracted from overnight cultures of *Sgg* grown in BHI using the E.Z.N.A. Bacterial RNA Kit (Omega). All RNA preparations were treated with DNase. cDNA was synthesized using a ProtoScript II First Strand cDNA Synthesis Kit (NEB). PCR primers are listed in Table 1. PCR reactions were performed using Taq polymerase and appropriate annealing temperatures.

**Table 1:** Oligonucleotides used in this study.

	Forward (5’-3’)	Reverse (5’-3’)
*esxA-esaA*	CTGAGGGAGCAACTTCAGTACGTG	CCACTATCTGCTGTACCACGAG
*esaA-essA*	GTCATCTTACTTTGATGGGCAATCAGC	CCCCATAAAAGATCGCTAAAAGACCAC
*essA-esaB*	GTGGGGCTAGTTTTGCTACCTACG	GTCCACTACACTAACTCCATCTCCTC
*esaB-essB*	CGCGTTGTGAATAAAGGTCTAG	CACGCGCTCATCTTTCG
*essB-essC*	GACGGATGCTTCGGGC	CATCCCCAAAAGAAAGCTGG
*essC-507*	GATACTGCTTTAATAGGGTTAAGAATG	CATCTTCATCTTTAAGCCCCTC
*507–508*	GAGGGGCTTAAAGATGAAGATG	GCCACTTCTGATGCCATCTGAC
*508–509*	GCACAATTTTTGACAGATATTCAAGGTC	GCTTCTCCTTGTAATGTATTTTGAAGTGTACTAAATTGCGC
*509–510*	GCGCAATTTAGTACACTTCAAAATACATTACAAGGAGAAGC	CTAGTTGTAGCCTTACCTTTTAGATTAGTAG
*510–511*	GTGACTTCAAACGCTAATAAGCTAG	GTCTCCAATTCTGTTTTCGCAC
*511–512*	GGAGCTTATGCGGCTGTGGATTGGGTAGAAG	CTGAAAAGCTACAATAAATTGGTAATAAGC
*512–513*	CGCTAAAATCACTTTTTATTATCAATAAGAATCAAG	CCCCCACCCTTGTAGTCAATGAGC
*Sgg_esxA*	TGAGGGAGCAACTTCAGTACG	GTGCATCTGTTTCTTCTAGCGT
*Sgg_essC*	TAACGCTTCTTGCTGGCTCA	GCTCTTTAGCTGGCGCATTG
*Sgg500*	GGAAAATGTTGATGTGGCTATCCTTGATGTGG	GTTGTTGGCGGATAATTTGTTAAGGATAGATGAGATG
*Sgg507*	ATGTCACAAGAATTATTACCGTTAGGATCTGTTGT	CCGATTTTTTTTATCTACAGGGATTTTTTCTTTAGTTCATT

### Deletion of *Sgg*T7SS^T05^

The mutagenesis system developed by Danne *et al*. [89] was used to delete *Sgg*T7SS^T05^. Briefly, the ~ 1kb region upstream of *Sgg_esxA* and the ~1kb region in the C-terminal portion of *Sgg*_*essC* were synthesized by Genscript and cloned into pUC57 (Genscript). The insert was then subcloned into a temperature sensitive conjugative plasmid pG1-*oriT*_TnGBS1_. The construct sequence was verified and introduced into *S*. *agalactiae* NEM316 by electroporation and then into *Sgg* strain TX20005 by conjugation under the permissive temperature. Erythromycin-resistant transconjugants were then cultured under a non-permissive temperature to select for single cross-over recombinants, followed by serial passage in antibiotic-free BHI and screening for double cross-over deletion mutants by PCR. Deletion was confirmed by PCR amplification of the regions spanning the deleted fragment and DNA sequencing of the PCR product, and by whole genome sequencing.

### Growth curve

Overnight cultures of *Sgg* were inoculated into fresh BHI broth at 1:100 dilution and grown at 37°C with shaking. Samples were taken at 0, 3, 6, 9, 12, and 24 hours, dilution plated, incubated for 24 hours and colonies enumerated.

### Western blot

For the detection of *Sgg*EsxA, TX20005 and TX20005*Δesx* were grown in BHI at 37°C with shaking for ~18 hours. Cultures were centrifuged, supernatants collected, filtered through a 0.2 μm filter, and concentrated using the Pierce™ Protein Concentrator PES, 3K Molecular Weight Cutoff. Bacterial pellets were washed and resuspended in PBS. Supernatants and bacterial suspensions were boiled in SDS loading dye and subjected to SDS-PAGE. Membranes were probed with anti-EsxA rabbit serum (1:100) (Pacific Immunological) and anti-FtsZ (1:250) antibodies (Abbexa, cat no. abx319936), followed by incubation with secondary antibodies conjugated to horse radish peroxidase (HRP) (1:3000). Blots were washed and immersed in Clarity Max ECL (Bio-rad). Images were acquired using a Fluorchem M chemiluminescent imager (Protein Simple).

Detection of β-catenin and PCNA was carried out as described previously [12]. Briefly, total cell lysates from HT29 cells cultured in the presence or absence of bacteria or CS were subjected to SDS-PAGE, transferred, and probed with antibodies against β-catenin (1:1000), PCNA (1:1000), and β-actin (1:1000) (Cell Signaling Technology), followed by incubation with HRP-conjugated secondary antibodies (1:3000). Band intensities were measured using ImageJ and normalized to that of β-actin.

### Animal experiments

Animal studies were performed in accordance with protocols approved by the Institutional Animal Care and Use Committee at the Texas A&M Health Science Center, Institute of Biosciences and Technology. Mice were fed with standard ProLab IsoPro RMH3000 (LabDiet). **(1) Colonization experiments.** This was performed as previously described [26] with slight modifications. Briefly, 6-week old A/J mice, sex matched (Jackson Laboratory), were treated with ampicillin at a concentration of 1 g/L in drinking water for 3 days and switched to antibiotic-free water 24 hours prior to administration of bacteria. *Sgg* was orally gavaged at a dose of 1 x 10^9^ CFU/mouse. Colons and fecal materials were collected at day 1, 3, and 7 post-gavage. Samples were homogenized in PBS in a TissueLyser (Qiagen), dilution plated onto Enterococcus Selective Agar (ESA) plates and incubated at 37°C for 24–48 hours to enumerate *Sgg* colonies, as previously described [76]. **(2) AOM-induced model of CRC.** This was performed as previously described [12] with slight modifications. Briefly, A/J mice were treated with 4 weekly i.p. injections of AOM (10 mg/kg body weight), followed by ampicillin in drinking water (1 g/L) for 7 days and switched to antibiotic-free water 24 hours prior to the first oral gavage with *Sgg*. Mice were orally gavaged with bacteria at ~ 1x10^9^ CFU/mouse or saline once per week for 12 weeks. Colons of mice were collected, cut opened longitudinally, and tumor number and size were recorded. Tumor burden was calculated as the sum of all tumor volumes per mouse. Visual evaluation of colons was carried out by a blinded observer.

### Statistical analysis

GraphPad Prism 8 was used for statistical analyses. Two-tailed unpaired *t*-test was used for pairwise comparisons to assess the significance of differences between two groups in cell proliferation assays, western blot analysis, and adherence assays. The non-parametric Mann-Whitney test was used to assess the significance of differences in the bacterial load and tumor burden in animal studies. Two-way ANOVA was used to analyze the bacterial growth curves. Ns, not significant; *, *p* < 0.05; **, *p* < 0.01; ***, *p* < 0.001; ****, *p* < 0.0001.

## Supporting information

S1 Table Raw and corrected read matrices for genome sequencing of TX20005. (DOCX)

S1 Fig Expression and purification of recombinant *Sgg*EsxA (rEsxA). The DNA sequence encoding full-length *Sgg*EsxA was codon optimized, synthesized by IDT as a gBlocks gene fragment, and then cloned into the pWL613a vector (a pET28b-based vector with an N-terminal 6His tag and a TEV protease cleavage site) which was linearized with BamHI and XhoI, thus producing a construct which expresses 6His-*Sgg*EsxA driven by a T7/*lac* promoter. The integrity of the resulting plasmid was confirmed by DNA sequencing. The construct was transformed into *E*. *coli* strain Rosetta2 (DE3) (Novagen) and protein expression was induced with 1 mM isopropyl 1-thio-β-D-galactopyranoside. Recombinant 6His-*Sgg*EsxA was purified using a HisTrap column (GE Healthcare), followed by a HiPrep 26/10 desalting column (GE Healthcare). The purified protein was digested with TEV to remove the His tag, and loaded onto a HisTrap 5 column to collect the flow-through, which was then further purified by size exclusion chromatography using a HiLoad 16/600 Superdex 200 pg column (GE Healthcare) (**A**). The purified tag-free rEsxA was examined via 4–20% gradient sodium dodecyl sulfate–polyacrylamide gel electrophoresis (SDS-PAGE) and Coomassie blue staining (**B**).
(TIF)

S2 Fig Deletion of *Sgg_esxA* to *Sgg_essC* does not affect the transcription of upstream or downstream genes. RNA was extracted from stationary phase TX20005 grown in BHI, treated with DNase and reverse transcribed. PCR was performed using primer pairs internal to the gene upstream of *Sgg_esxA* (*Sgg*500) and the gene downstream of *Sgg_essC* (*Sgg*507) (Table 1). Genomic DNA (gDNA) from TX20005 was used as a positive control for PCR, and RNA without reverse transcriptase treatment (no RT) was used as a control for possible DNA contamination.
(TIF)

S3 Fig Western blot analysis of culture supernatants (CS) from TX20005 and TX20005Δ*esx*. CS was prepared from *Sgg* grown in BHI broth with shaking for ~ 18 hours and analyzed as described in the Materials and Methods section. An equivalent of ~ 0.5 ml of overnight cultures was loaded onto an SDS gel. The membrane was probed with anti-EsxA antiserum followed by HRP-conjugated secondary antibodies. Purified rEsxA was used as a control for the protein.
(TIF)

S4 Fig Growth of TX20005 and TX20005Δ*esx* in the cell culture conditions with trimethoprim. Stationary phase TX20005 and TX20005Δ*esx* was inoculated into the appropriate cell culture media (time 0) and incubated following the procedure described for cell proliferation assays. Samples were taken at 0 hour, immediately after the addition of trimethoprim (6 hour), and at the end of the incubation (24 hour). Bacterial titer was determined by dilution plating of the culture media onto tryptic soy agar and incubating for 24–48 hours.
(TIF)

S5 Fig *Sgg* burden in the proximal and distal portion of the colon. Colons collected at day 3 (**A**) and 7 (**B**) post bacterial gavage from the colonization experiment were separated into proximal and distal portions, weighed, homogenized and plated onto Enterococcus Selective Agar plates to enumerate *Sgg* bacteria. Data shown are the mean ± SD (n = 5/group).
(TIF)
