# Supplementary material for: A type VII secretion system of Streptococcus gallolyticus subsp. gallolyticus contributes to gut colonization and the development of colon tumors
Source: PLoS Pathog. 2021 Jan 6;17(1):e1009182. doi: 10.1371/journal.ppat.1009182 (PMC7815207; doi:10.1371/journal.ppat.1009182)
Supplement: S1 Table — (DOCX) [file ppat.1009182.s001.docx]

**S1 Table. Raw and corrected read matrices for genome sequencing of TX20005.**

|  | **Total Bases** | **Total Reads** | **Smallest** | **Largest** | **Average** | **Median** | | **N50** |
| --- | --- | --- | --- | --- | --- | --- | --- | --- |
| **Raw Reads** | 4288335939 | 316535 | 103 | 98731 | 13547 | | 22191 | 18313 |
| **Post Error Correction** | 398572620 | 39767 | 5778 | 46056 | 10022 | | 11815 | 8945 |
