# Supplementary figures and images for: A type VII secretion system of Streptococcus gallolyticus subsp. gallolyticus contributes to gut colonization and the development of colon tumors
Source: PLoS Pathog. 2021 Jan 6;17(1):e1009182. doi: 10.1371/journal.ppat.1009182 (PMC7815207; doi:10.1371/journal.ppat.1009182)

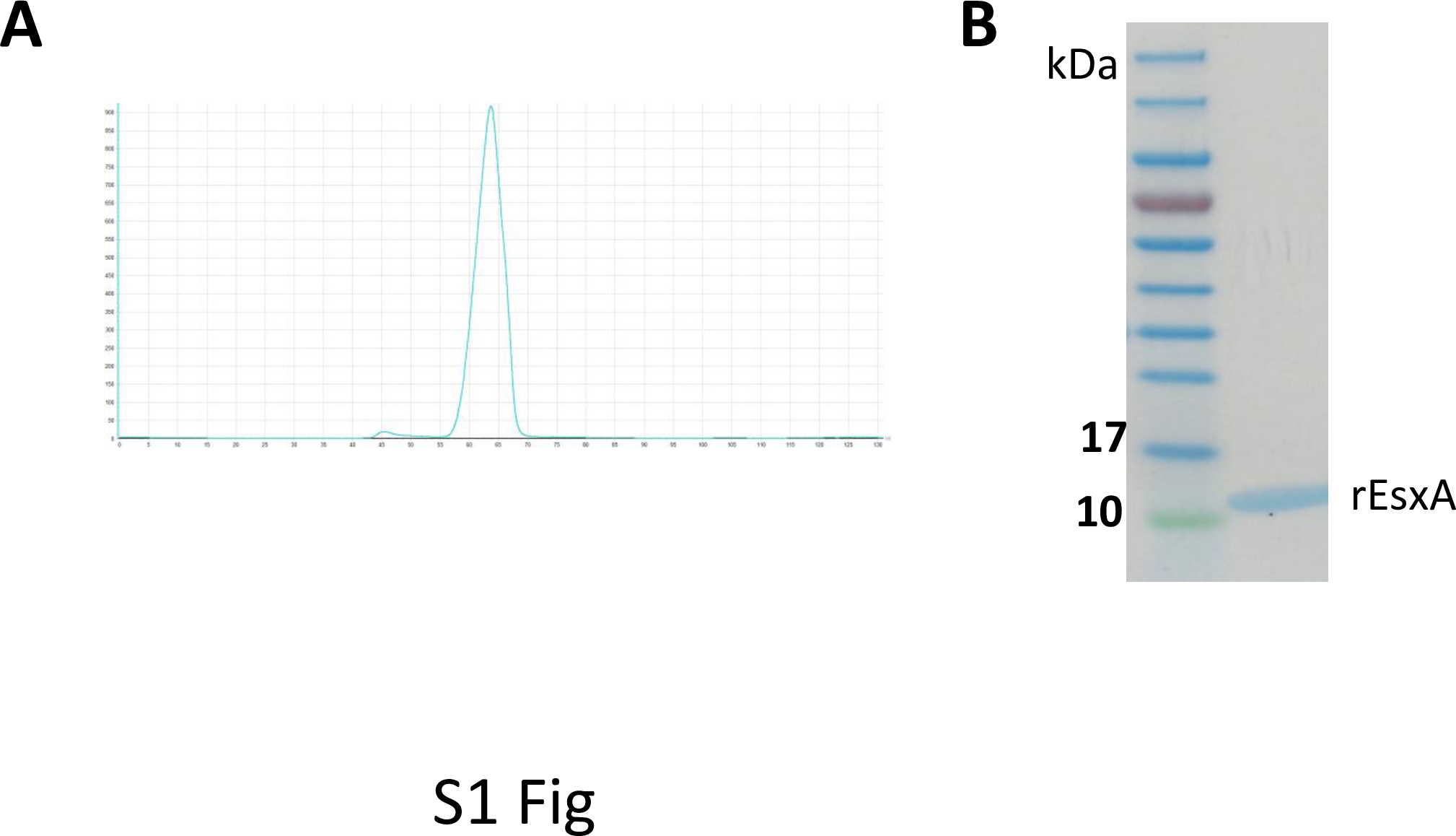

Supplement: S1 Fig — The DNA sequence encoding full-length SggEsxA was codon optimized, synthesized by IDT as a gBlocks gene fragment, and then cloned into the pWL613a vector (a pET28b-based vector with an N-terminal 6His tag and a TEV protease cleavage site) which was linearized with BamHI and XhoI, thus producing a construct which expresses 6His-SggEsxA driven by a T7/lac promoter. The integrity of the resulting plasmid was confirmed by DNA sequencing. The construct was transformed into E. coli strain Rosetta2 (DE3) (Novagen) and protein expression was induced with 1 mM isopropyl 1-thio-β-D-galactopyranoside. Recombinant 6His-SggEsxA was purified using a HisTrap column (GE Healthcare), followed by a HiPrep 26/10 desalting column (GE Healthcare). The purified protein was digested with TEV to remove the His tag, and loaded onto a HisTrap 5 column to collect the flow-through, which was then further purified by size exclusion chromatography using a HiLoad 16/600 Superdex 200 pg column (GE Healthcare) (A). The purified tag-free rEsxA was examined via 4–20% gradient sodium dodecyl sulfate–polyacrylamide gel electrophoresis (SDS-PAGE) and Coomassie blue staining (B). (TIF) [file ppat.1009182.s002.tif]

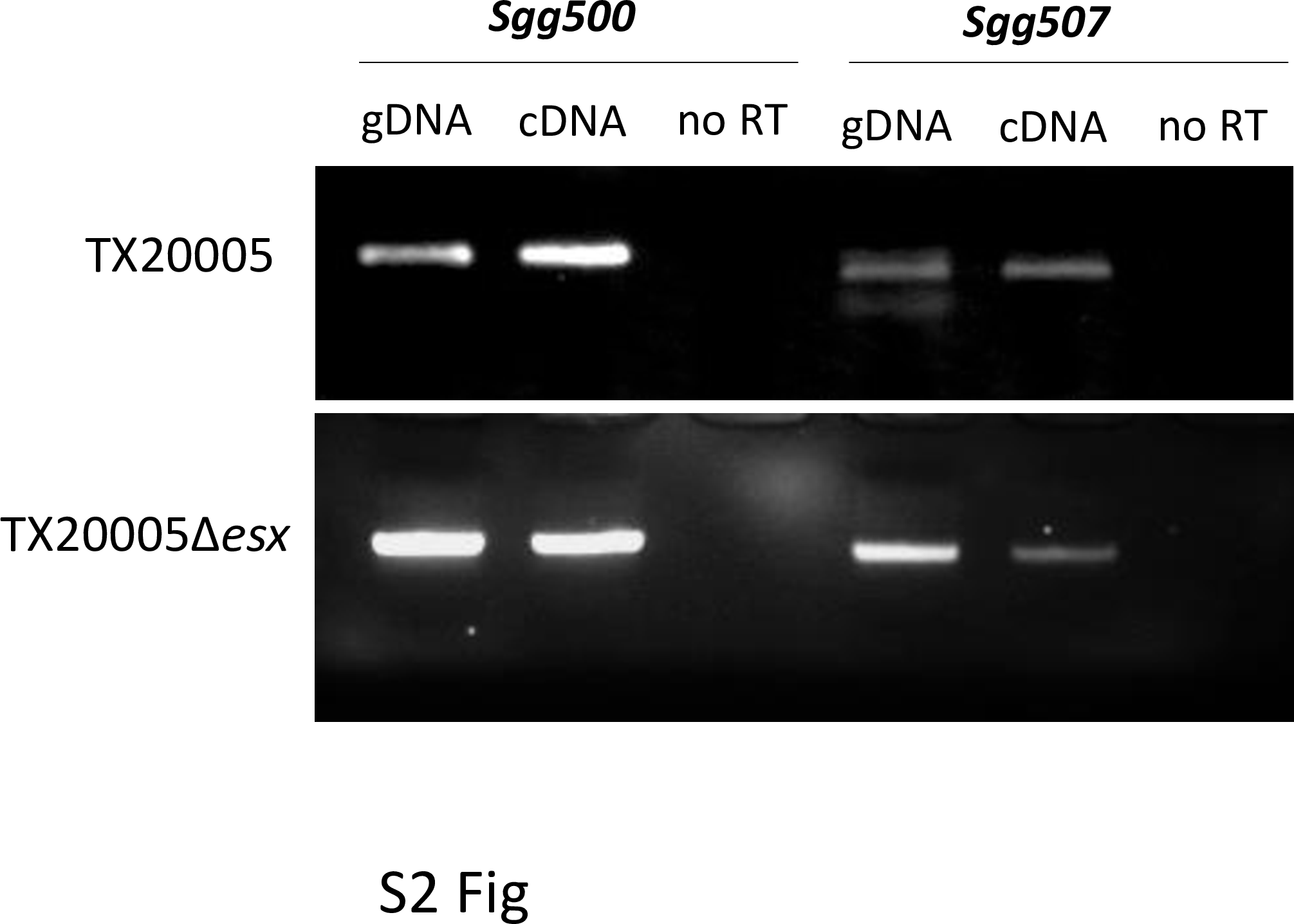

Supplement: S2 Fig — RNA was extracted from stationary phase TX20005 grown in BHI, treated with DNase and reverse transcribed. PCR was performed using primer pairs internal to the gene upstream of Sgg_esxA (Sgg500) and the gene downstream of Sgg_essC (Sgg507) (Table 1). Genomic DNA (gDNA) from TX20005 was used as a positive control for PCR, and RNA without reverse transcriptase treatment (no RT) was used as a control for possible DNA contamination. (TIF) [file ppat.1009182.s003.tif]

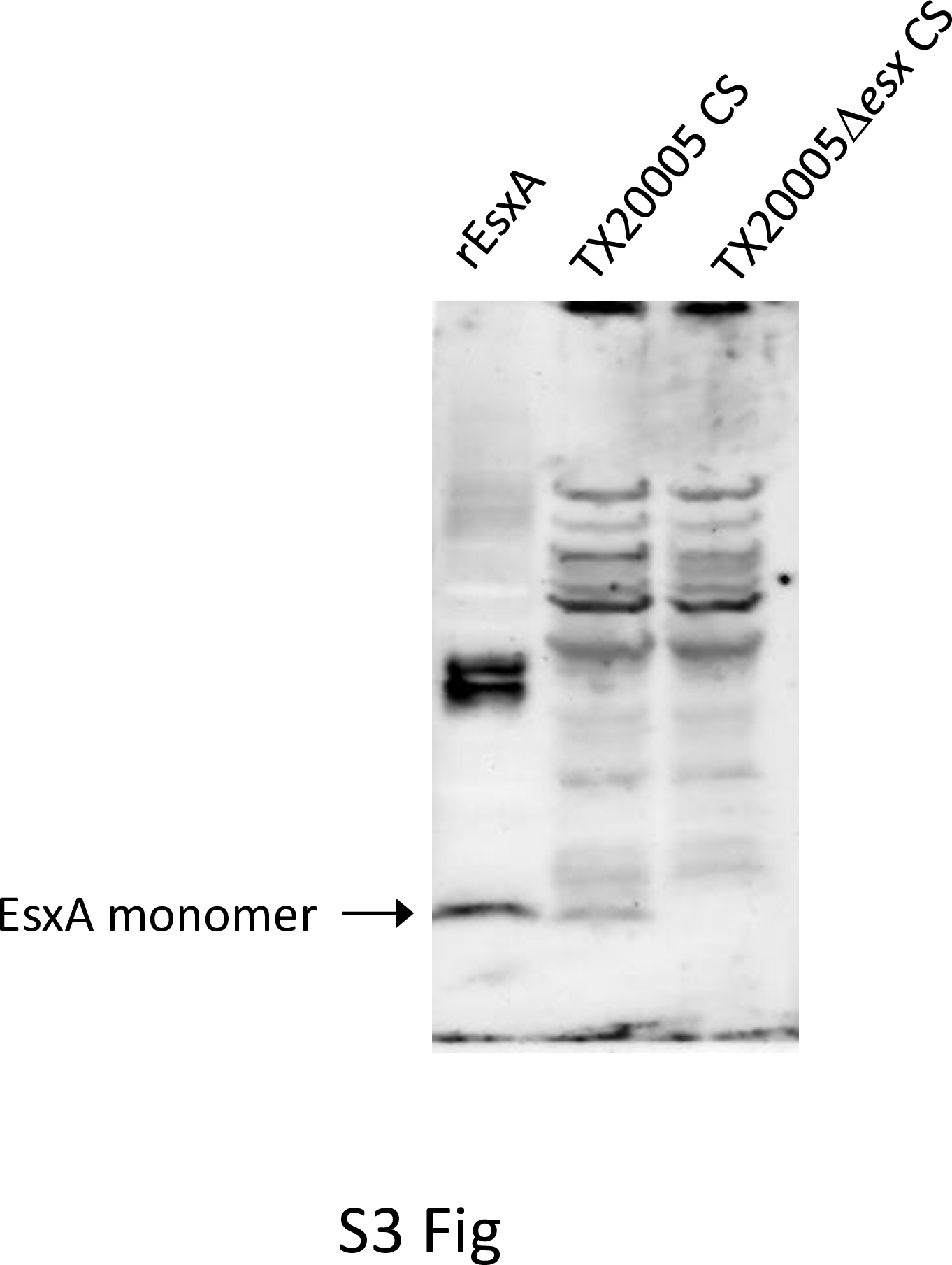

Supplement: S3 Fig — CS was prepared from Sgg grown in BHI broth with shaking for ~ 18 hours and analyzed as described in the Materials and Methods section. An equivalent of ~ 0.5 ml of overnight cultures was loaded onto an SDS gel. The membrane was probed with anti-EsxA antiserum followed by HRP-conjugated secondary antibodies. Purified rEsxA was used as a control for the protein. (TIF) [file ppat.1009182.s004.tif]

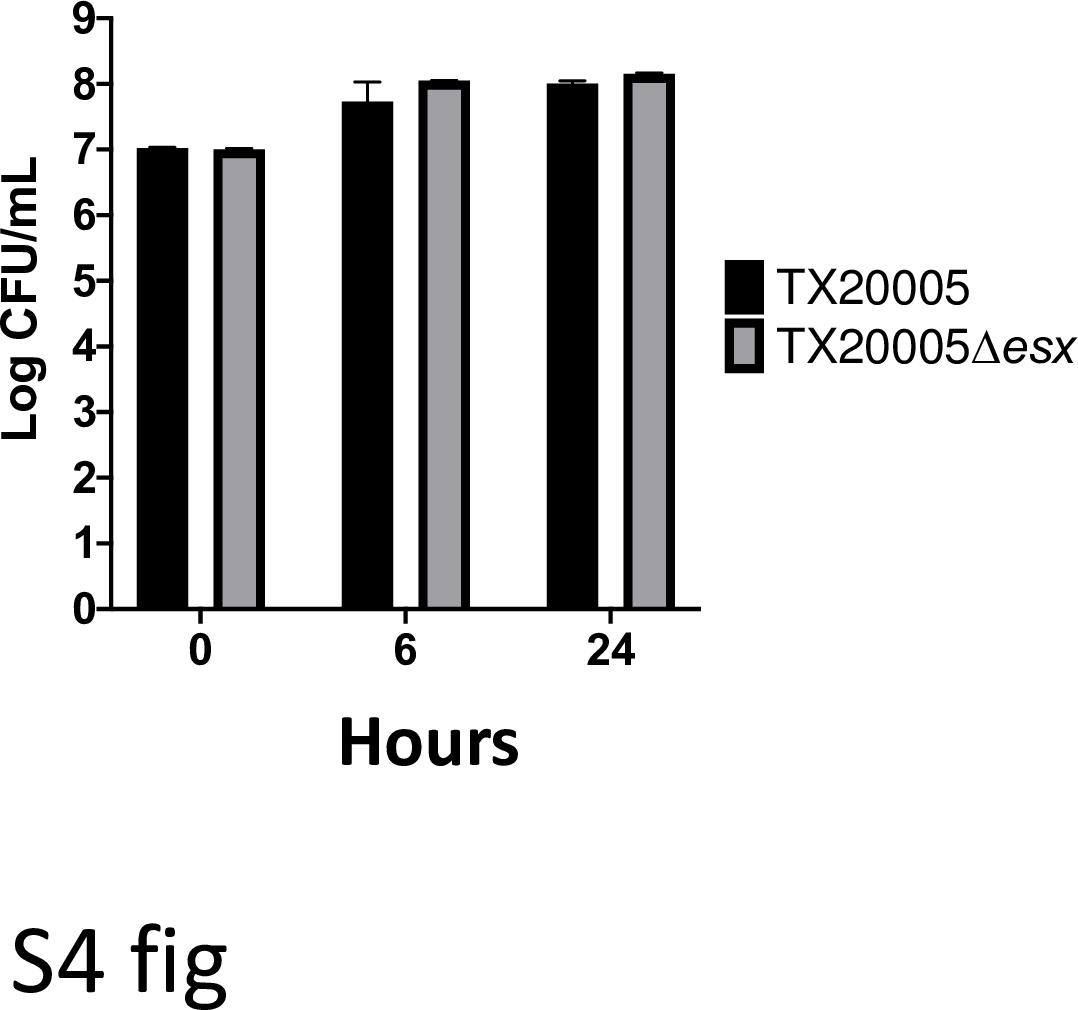

Supplement: S4 Fig — Stationary phase TX20005 and TX20005Δesx was inoculated into the appropriate cell culture media (time 0) and incubated following the procedure described for cell proliferation assays. Samples were taken at 0 hour, immediately after the addition of trimethoprim (6 hour), and at the end of the incubation (24 hour). Bacterial titer was determined by dilution plating of the culture media onto tryptic soy agar and incubating for 24–48 hours. (TIF) [file ppat.1009182.s005.tif]

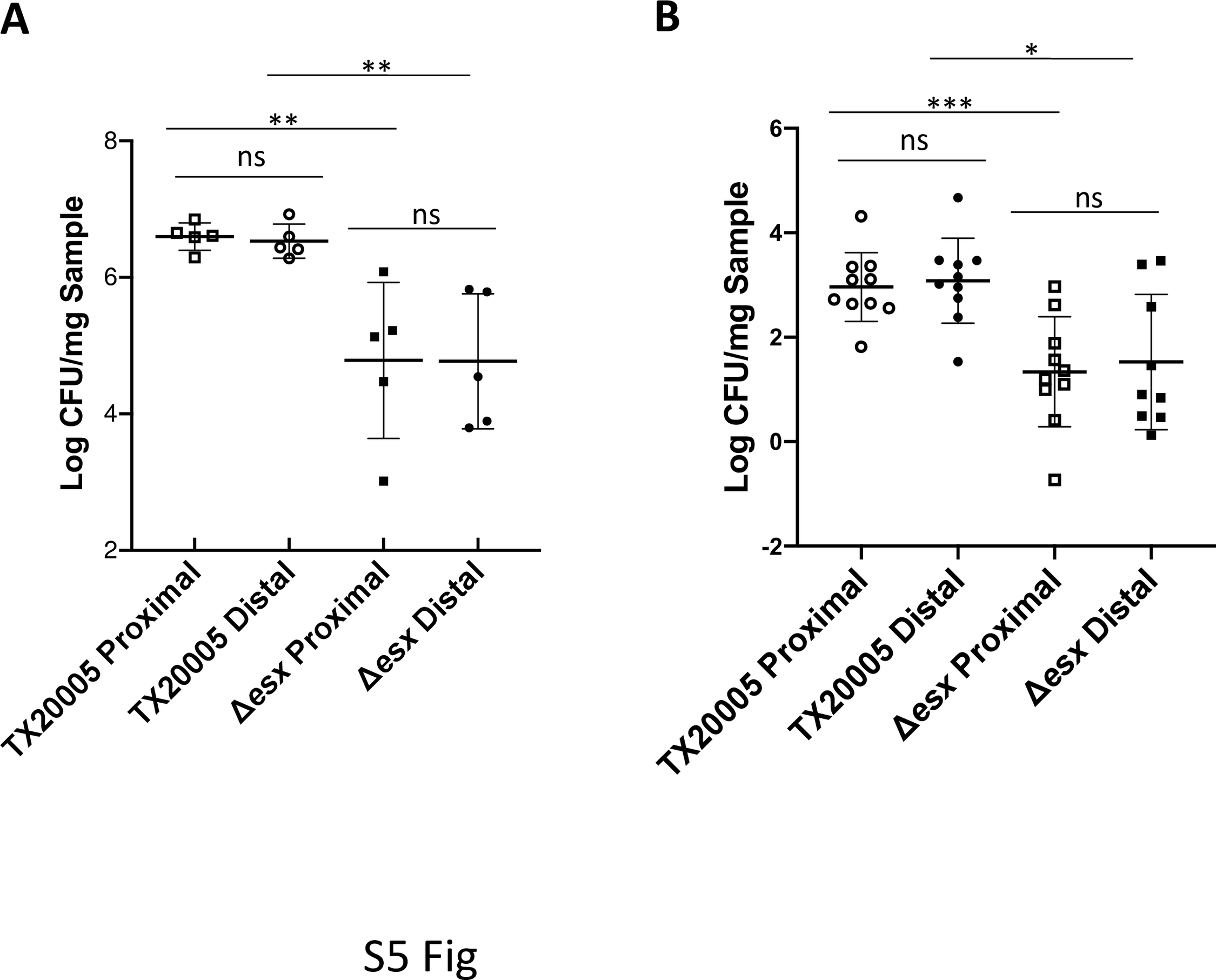

Supplement: S5 Fig — Colons collected at day 3 (A) and 7 (B) post bacterial gavage from the colonization experiment were separated into proximal and distal portions, weighed, homogenized and plated onto Enterococcus Selective Agar plates to enumerate Sgg bacteria. Data shown are the mean ± SD (n = 5/group). (TIF) [file ppat.1009182.s006.tif]
